# Pragmatic considerations and approaches for measuring staff time as an implementation cost in health systems and clinics: key issues and applied examples

**DOI:** 10.1186/s43058-022-00292-4

**Published:** 2022-04-15

**Authors:** Amy G. Huebschmann, Katy E. Trinkley, Mark Gritz, Russell E. Glasgow

**Affiliations:** 1 Division of General Internal Medicine, University of Colorado Anschutz Medical Campus (CU-AMC), 12631 E. 17th Ave., Mailstop B180, Aurora, CO 80045 USA; 2 CU-AMC Adult and Child Center for Outcomes Research and Delivery Science (ACCORDS), Aurora, CO USA; 3 CU-AMC Ludeman Family Center for Women’s Health Research, Aurora, CO USA; 4 CU-AMC Skaggs School of Pharmacy and Pharmaceutical Sciences Department of Clinical Pharmacy, Aurora, CO USA; 5 University of Colorado Health (UCHealth) System Department of Clinical Informatics, Aurora, CO USA; 6 CU-AMC, Division of Health Care Policy and Research, Aurora, CO USA; 7 CU-AMC Department of Family Medicine, Aurora, CO USA

**Keywords:** Costing, Costs and cost analysis, Implementation, Time-driven activity-based costing, Program delivery, Health workforce; Staff time estimation

## Abstract

**Background:**

As the field of implementation science wrestles with the need for system decision-makers to anticipate the budget impact of implementing new programs, there has been a push to report implementation costs more transparently. For this purpose, the method of time-driven activity-based costing (TDABC) has been heralded as a pragmatic advance. However, a recent TDABC review found that conventional methods for estimating staff time remain resource-intensive and called for simpler alternatives. Our objective was to conceptually compare conventional and emerging TDABC approaches to measuring staff time.

**Methods:**

Our environmental scan of TDABC methods identified several categories of approaches for staff time estimation; across these categories, staff time was converted to cost as a pro-rated fraction of salary/benefits. Conventional approaches used a process map to identify each step of program delivery and estimated the staff time used at each step in one of 3 ways: (a) uniform estimates of time needed for commonly occurring tasks (self-report), (b) retrospective “time diary” (self-report), or (c) periodic direct observation. In contrast, novel semi-automated electronic health record (EHR) approaches “nudge” staff to self-report time for specific process map step(s)—serving as a contemporaneous time diary. Also, novel EHR-based automated approaches include timestamps to track specific steps in a process map. We compared the utility of these TDABC approach categories according to the 5 R’s model that measures domains of interest to system decision-makers: relevance, rapidity, rigor, resources, and replicability, and include two illustrative case examples.

**Results:**

The 3 conventional TDABC staff time estimation methods are highly relevant to settings but have limited rapidity, variable rigor, are rather resource-intensive, and have varying replicability. In contrast to conventional TDABC methods, the semi-automated and automated EHR-based approaches have high rapidity, similar rigor, similar replicability, and are less resource-intensive, but have varying relevance to settings.

**Conclusions:**

This synthesis and evaluation of conventional and emerging methods for staff time estimation by TDABC provides the field of implementation science with options beyond the current approaches. The field remains pressed to innovatively and pragmatically measure costs of program delivery that rate favorably across all of the 5 R’s domains.

Contributions to the literature

- The decision to adopt, implement, and sustain an evidence-based program is heavily influenced by cost. Often, staff time is a major driver of costs for a program.
- Time-driven activity-based costing is widely used to measure implementation costs of a program, but current approaches to staff time estimation remain resource-intensive.
- We reviewed conventional (uniform estimate, time diary, direct observation) and emerging electronic health record-based approaches to measure staff time used and compared these categories of approaches based on relevance to stakeholders, rapidity, rigor, resource requirements, and replicability.
- The electronic health record provides the field of implementation science with new semi-automated and automated opportunities to assess time that can be rapid, rigorous, replicable, and low-burden in terms of resources required, but their relevance to a given setting/project may vary.

## Background

The field of implementation science (IS) has made great progress in identifying critical approaches to translate evidence-based programs (EBP) into practice [1, 2]. Despite this progress to guide the implementation of an EBP into a given health setting, persistent dissemination challenges include: (1) inconsistent “scaling up” to varied settings within a health system, (2) “scaling out” across different health systems remains rare, and (3) sustainment of these changes is difficult. When system-level decision makers lack information on the cost of implementing and sustaining EBPs, it deters dissemination and sustainment [3–5]. Some IS frameworks, including the Veterans Administration QUality Enhancement Research Initiative (VA QUERI) roadmap [6], seek to guide scaling up EBPs by considering different types of implementation costs within the following project phases: (1) pre-implementation, (2) implementation, and (3) sustainment [6]. During the pre-implementation and implementation phases, key cost considerations are as follows: (1) “capacity” for delivering the EBP, including the cost of staff time for both EBP delivery and the implementation strategy of staff training, and (2) comparing the costs of alternate implementation strategies. In the sustainment phase, the focus shifts to estimate the staff time needed to continue to deliver the EBP and implementation strategies, as well as other ongoing system costs such as program materials [5].

Recent reviews of cost assessment approaches for IS and improvement science have specified the need to track the staff time required for both EBP delivery and for implementation strategies used [5, 7–9]. Drilling down into the staff time costs for both of EBP delivery and implementation strategies is important because (1) staff time is a major source of costs for EBP delivery; (2) staff time is a costly element of certain implementation strategies, such as technical assistance and training; and (3) assessment of other types of costs, such as program materials, are more straightforward to track. The method of time-driven activity-based costing (TDABC) has been heralded as a relatively pragmatic approach to estimate the staff time required for these different tasks; accordingly, the use of TDABC in IS research has accelerated recently [3–5].

As developed by Kaplan et al. [3], TDABC methods specify costs across several steps of EBP implementation. A central aspect of TDABC is to create a process map that allocates the time for each staff actor to complete each process map step, inclusive of both EBP delivery and implementation strategies used [5]. However, a recent review of TDABC by Keel et al. [4] concluded that current approaches for staff time estimation in each step of a TDABC process map remain resource-intensive and called for the development of simpler and more rapid approaches with less resource burden [4]. Accordingly, the field would benefit from more pragmatic staff time estimation approaches, with balanced attention to rigorous and reliable data collection methods [10].

Thus, there is a need to contrast the conventional methods of staff time estimation with some novel and emerging electronic health record (EHR)-based methods that could address some of the current challenges. The purpose of this brief methodology report is to compare distinct categories of conventional and emerging TDABC approaches to staff time estimation according to the 5 R’s model [11] for pragmatism that measures domains of interest to researchers and health system decision-makers: relevance, rapidity, rigor, resources, and replicability. In contrast to recent reviews and commentaries that only considered conventional approaches to staff time estimation by TDABC [4, 5, 7–10], this paper also considers innovative automated and semi-automated EHR-based approaches, and compares these different approaches on each of the 5 R’s domains. We also provide two illustrative case study examples that delineate why different staff time estimation approaches may be selected. This environmental scan of emerging pragmatic methods for staff time estimation provides the field of IS with options beyond the current standards of observation or asynchronous reporting, and presses the field to identify additional non-intrusive, real-time approaches to assessing implementation costs.

## Methods

We conducted an environmental scan, including a literature search, for articles measuring the cost of staff time to implement healthcare-related EBPs. We searched PubMed using the following search terms: (“implementation cost” or “time-driven activity-based cost” or “micro-cost”) and (“health*” or “clinic*”). The literature search was limited to articles in English over the past 5 years. Articles’ references were hand-searched for additional articles. We also queried an online community of EHR users (Epic UserWeb) and colleagues with experience in EHR approaches to time capture: a clinical informatics nurse research scientist and two physician informaticists.

Our intent was not to conduct a systematic review, but to use this environmental scan to identify existing categories of staff time estimation approaches, and to compare the relative pragmatism of these approaches using the 5 R’s model perspective [11] (Table 1). While not exclusive to IS, the 5 R’s was selected because it was developed to increase the pragmatism of health research and is an accepted model of pragmatic health research domains [11–13]. The 5 R’s framework emphasis on relevance, rigor, and replicability are complementary with the approach that Cidav et al. took to track TDABC according to the Proctor et al. framework [12] by specifying who/what/when/how often/for how long an individual delivers an implementation strategy, but also adds an explicit emphasis on rapid/low resource burden approaches [5].

**Table 1:** Application of the 5 R’s to evaluate cost assessment approaches

5 R’s model issue	How the issue was applied to evaluate the cost assessment approaches
Relevant	Is it understandable to decision makers? Does it address decision makers’ informational needs?
Rapid and recursive	Does it provide information to decision makers when needed? Does it allow iterative evaluation to facilitate refinement of the implementation strategy?
Rigorous	How accurate and reliable is it at estimating actual time, including ‘hidden’ costs?
Resources required	How labor-intensive is the approach? Does it require dedicated personnel?
Replicable	How likely can it be reproduced by another party? Is it generalizable across different types of settings and programs?

Approaches to staff time estimation were evaluated from the perspective of system-level decision makers. Decision makers did not participate in the review process, but we considered their perspective on how a new EBP would impact their budget. Using a content analysis approach, two authors (KT and AH) reviewed the distinct approaches to staff time estimation in each article and placed them in categories named for common time capture terms [14]. We evaluated these categories of approaches from the 5 R’s model perspective [11] (Table 1), providing more favorable ratings if they (1) rated high in relevance to stakeholders, rapidity, and recursiveness, rigor, and replicability and (2) required few resources.

## Results

From our environmental scan, we identified several categories of approaches to estimate the staff time spent implementing EBPs as a part of TDABC [4, 5, 7, 15]. These categories of staff time estimation approaches are applicable to EBP program delivery by managers, supervisors, and staff, as well as implementation strategies (e.g., training to deliver the EBP, and other time spent preparing for the program); time spent evaluating the program; and indirect time costs of the program on patients and care givers [5, 7, 15].

With the caveat that the approaches used to capture staff time were not always clearly described in our literature search, and a given study sometimes used more than one category of staff time estimation approach in concert [4], the most common conventional approaches reported were self-report using a time-reporting template or “time diary” [16–18] and uniform self-report estimates of time spent on certain activities [5, 7, 19–21]. Some studies also reported a category of direct observation [22–24]. Using our pre-specified search terms, we found one study reporting use of an automated EHR-related approach [23]. Our broad environmental scan also identified other articles using semi-automated or automated EHR-based approaches for staff time estimation, including recommendations for their use and reporting [25, 26]. Our summary of these TDABC categories of approaches are summarized in Table 2 from a 5 R’s model perspective.

**Table 2:** Comparison of current categories of TDABC approaches to staff time estimation

Approach	Brief description	Benefits and Challenges mapped to Domains of 5 R’s framework
**Self-report approaches**		
Uniform estimates of time for tasks [5, 7]	- An average time is assessed for common tasks - Often used in concert with a process map to identify all tasks and the task frequency/actors - Can be self-tracked or an evaluation team member prompts individuals in each role to understand the time required for common tasks	**• Relevance** is high to implementers—these tasks are part of the existing process **• Rapidity** is low-to-moderate—some delays to initially estimate data, but data collection is fairly rapid **• Rigor** is moderate—recall bias is minimized for common tasks, but this may be a bigger problem for rare tasks. Systems use this approach more for common tasks **• Replicability** is moderate—other systems likely use different process maps, so this may not be fully replicable **• Resources** required are moderate for research assistants or quality improvement teams—both to ensure process map is accurate and to send surveys and compile survey results. This poses a challenge to continue tracking in a sustainment period
Retrospective time diary [16]	- Time diary may include a template or time card that tracks time spent in different aspects of a project - Recorded retrospectively (e.g., time spent in last week or last month), but can be real time - Either self-tracked or may need to be interviewed or prompted to complete	**• Relevance is high**—specific to the time spent on a relevant task for implementers **• Rapidity** is low-to-moderate—there are often delays to compile these self-report data, but templates are fairly quick to complete. Occasionally, these may be triggered automatically **• Rigor** is moderate, but recall bias is minimized for common tasks and by more frequent assessments with shorter recall periods (e.g., recall over 1 week is more rigorous than recall over a period of 1-3 months); as a negative, when using a time card type of approach, there is a more limited ability to capture variation of task type as compared to the uniform estimates of time for task approaches **• Replicability** is high—others may replicate this approach **• Resource**s required are moderate—must be prompted to complete time diary (other than when it is triggered automatically) and compile survey results - thus at-risk to continue this in sustainment period
**Third party observer approaches**		
Direct observation [22]	- Specific observation template for 3rd-party to document time spent in different aspects of project and by different staff members	**• Relevance** is high to implementers—these tasks are part of the existing process **• Rapidity** is low—this is a time-consuming process to observe and compile data, particularly for low-frequency events **• Rigor** is high due to direct observation—used as a “gold standard” comparison [27] **• Replicabilit**y is high—others may replicate this approach **• Resources** required are high, and this is particularly challenging for low-frequency events due to large periods of “down time” for observers
**Semi-automated approaches**		
Contemporaneous time diary embedded within electronic health record [26]	- Staff have a designated field to complete within the standard note used for delivering the EBP - Time template completed at the same time as delivery of each instance of the EBP	**• Relevance** is high to implementers **• Rapidity** is moderate-to-high—data are available immediately but staff or research assistants may still need to compile **• Rigor** is moderate-to-high—recall bias is mitigated by completing the template at time of EBP delivery but there may still be inconsistent recall of time spent, particularly if differentiating between time spent on > 1 implementation strategy (e.g., time spent preparing, time spent with patient) **• Replicability is moderate-to high**—many EHRs may replicate this approach **• Resources** required are low-to-moderate, low if report may be auto-generated and requires limited technical support/staff time to set up
**Automated electronic health record (EHR) approaches**		
Time captured in real time by EHR or integrated software [23]	- Time spent completing certain EHR activities, such as a specific encounter type, is tracked using objective time stamps - What is tracked, how it is tracked, and data retrieval methods are specific to the EHR vendor and local system	**• Relevance** is moderate to implementers—may not capture a specific task as well as the other approaches listed **• Rapidity** is high if data are available immediately—**rapidity** is moderate if need to rely on vendor or local IT staff to run report at pre-specified intervals **• Rigor** can be high if multitasking is minimal/or if configured to stop recording if idle **• Replicability** is high within a system—may be difficult for other health systems to implement the same assessment program with a different EHR vendor **• Resources** required are low for data collection, may be moderate-to-high to set up

### Self-report/observation categories

We identified numerous articles using conventional self-report or observation approaches to estimate staff time [5, 7–9, 28]. As described above, these began with a process map to identify each step of EBP delivery and then estimated the staff time required at each step of the process map using one of these approaches: (a) uniform estimate of time needed for a commonly occurring task, (b) retrospective self-report in “time diary”, or (c) periodic direct observation. However, these approaches are somewhat resource-intensive, especially observation. Further, using these approaches, costs may not be feasible to capture during the sustainment phase when there are no grant funds to support observations and/or compilation of self-report data.

### Automated/semi-automated EHR-based approaches

For programs in settings that have EHRs, recent approaches have emerged to automate the data collection partly or fully. Semi-automated approaches may include hard stops built into a specific EHR note type that “nudge” a user to input their time—this essentially embeds a contemporaneous time diary into the note. Incorporating a contemporaneous time diary into the clinic note allows staff to review their charting to guide their time estimate reported and may lessen recall bias by completing the time diary in “real-time.” In contrast, fully automated approaches require no action by the staff. Seven categories of fully automated EHR-based approaches to staff time estimation are possible. These 7 categories are not mutually exclusive and include time spent: (1) documenting care provided—including the time spent within specific types of encounters, such as time spent documenting an anticoagulation visit encounter in the second case example below, (2) time spent placing or refilling prescriptions, (3) managing the EHR inbox including patient messages, (4) managing orders as part of the team, (5) providing direct patient care, (6) work during scheduled work hours, and (7) work outside of scheduled work hours [25].

With automated approaches, data are collected in real time (e.g., by the EHR), which avoids recall bias and reduces the resource burden. However, depending on the EHR vendor or other software used to track time, there are limitations in what activities can be tracked, the accuracy of the tracking estimates, and the timeliness of retrieving the data. For example, when clinicians are multitasking and leave EHR windows open, time estimates may be inflated. As it relates to relevance, if the encounter type that is tracked is not specific to the EBP and it also captures other tasks, it may not be fully relevant and the rigor of measurement is decreased. Regarding resources, some automated EHR-based approaches require assistance from the EHR vendor and/or local analysts/informaticists at the outset to determine what data to collect and how to access them. In addition, although collected in real time, the data may not be accessible in real time—data access may also require help from the EHR vendor or a local analyst/informaticist. After the initial set up, there are some benefits to EHR approaches, notably that when programs reach a sustainment phase [3], an automated method that was set up in the EHR can continue to provide reports of the staff time needed for a certain type of clinical encounter.

### Case examples

To further illustrate the tradeoffs of these different approaches, we provide an overview of the approaches employed in two real-world research case examples [26]. In the first case, the study used both self-report and semi-automated approaches for capturing time spent, whereas in the second case, the authors used both automated approaches and direct observation to develop a complete workflow process map and to validate the automated timing calculations. In Table 3, we provide the rationale for the approach selected in each case example, and at least one alternative approach that could have been used.

**Table 3:** Rationale for use of specific TDABC approaches in the case example pilot trials

Domain of implementation cost assessed [7]	Actual approach used to capture costs	Rationale for this approach	Alternative cost capture approaches *(problems with this alternative)*
Case example 1: type 2 hybrid trial of physical activity coaching in primary care [26]			
Training (coach time)	*Self-report*: retrospective time diary	The coaches’ employer received time diary data to track the time spent on this project, in order to allocate a proper portion of their salary to the research grant	*Self-report*: uniform estimates of time for tasks *(insufficient detail for the coaches’ employer’s goal to charge for the hours that each coach spent on the project)*
Delivering intervention coaching content (coach time)	*Semi-automated*: contemporaneous time diary embedded within the electronic health record (EHR, Epic Systems)	The PI captured the time each coach spent delivering the program, in order to compare the hours of coach time by certain patient demographic characteristics (e.g., age, gender, insurance type)	*Automated*: EHR estimate of time spent during this type of research encounter *(grant funds were insufficient to pay the health informatics team to create a specific research encounter type for this project)*
“Hidden” costs of preparing for the program—including technical assistance with transmitting data between patients and clinics (research assistant time)	None	At the outset of the grant, it was not clear that the research assistant would need to support patients and serve in the role of a “technician” to support data-sharing between patients and clinics	*Automated*: EHR estimates of time spent during this type of research encounter *(as noted above, grant funds were insufficient for this; in addition, it would not fit well with the research assistants’ work flow to document this technical assistance in study-specific documents rather than the EHR)*
Case example 2: Population-based cross-sectional study paired with observation of anticoagulation clinic processes [23]			
Delivering intervention (time for pharmacist, registered nurse, and clerical staff	Combination of automated and observation methods *Observation*: workflow process map *Automated*: a proprietary internal database (Workload Management Reporting system) estimated the time spent in each step of the process map	The proprietary database provided an accurate way to assess costs at each step of the process map	*Observation alone*: The authors also used direct observation to validate a subset of the automated cost capture timings derived from the system (*sole use of observation methods would have resulted in far fewer data points than the combination of observation and automated approaches*)

The first case example is from a pilot type 2 hybrid implementation/effectiveness trial studying the delivery of an evidence-based physical activity coaching intervention in a primary care clinic [26]. Staff time costs included the following: (1) an implementation strategy of training existing staff to serve as coaches; (2) time spent delivering the 6 intervention telephone calls to each patient, and (3) time for the implementation strategy of coaches providing technical assistance to patients to share their physical activity data (FitBit©). Approaches to capture time varied across the different elements of the program (Table 3). For time spent training, a conventional self-report time diary was used per the staff employer’s preference, in order to allocate the time spent to the research grant for this one-time session. To optimally capture the time spent in each counseling session, a semi-automated EHR-based approach was used to avoid recall bias: a brief, required contemporaneous time diary was embedded within the behavioral coaching note template in the EHR (Epic Systems). This embedded time collection template can easily be replicated in Epic Systems and other commonly used EHRs by creating a “required field” for time that must be documented before closing the note. In contrast to an alert that fires and interrupts workflow, this approach only nudges staff if the template was left incomplete when signing the encounter. During the pre-implementation phase, the coaches noted this approach fit their workflow and was minimally burdensome.

The second example is a program evaluation of the staff costs of delivering care at an anticoagulation clinic for various phenotypes of patients—those who needed minimal adjustment to their treatment regimen and those who needed frequent adjustments [23]. As they sought to compare variable costs across patients in the existing anticoagulation clinic where baseline training had already occurred, the authors did not assess staff training costs. Instead, they used direct observation to detail a process map of each step in the workflow for a patient to engage with the anticoagulation clinic staff. This included multiple steps for in-person visits, from the time of check-in until the time of check-out, and the time spent by nurses and pharmacists between in-person clinic visits. Using a proprietary internal database, the authors captured automated data for the time spent by *each member of the clinical team* in each step of the process map workflow. They also used a subset of direct observation assessments to validate these automated measurements of staff time. Using TDABC, they calculated the costs of the staff time in each step of the workflow, and then differentiated the costs for patients who were well-controlled and not well-controlled. Although this internal database was proprietary to their system, other EHRs, including the Epic Systems EHR [25], also have this capacity to track staff time.

## Discussion

This brief methodologic commentary compares several approaches to capturing the portion of implementation costs related to staff time—an important element of implementation according to the VA QUERI Roadmap [6] and other IS process models. In particular, approaches to capturing staff time are critical to transparently report to system decision-makers the time required to implement and sustain a program. Overall, the comparison of these approaches in Table 2 may be considered as a balance of data optimization (i.e., rigorous/reliable) and efficiency in terms of a rapid return of relevant findings with low-resource requirements. In terms of the rigor/reliability, the observational approaches are most accurate, followed by the automated and semi-automated EHR-based approaches, and then the retrospective time diary approaches that are particularly prone to recall biases. In terms of efficiency, the semi-automated/automated EHR approaches stand out for their rapidity and for the limited resources needed after their initial set-up, followed by self-report. Observational approaches are the slowest and most time-consuming.

It is interesting to further consider the relative merits of these approaches from the VA QUERI Roadmap perspective which dictates that estimates of staff time are most critical to assess in the Sustainment phase. Conventional self-report time diary and observational approaches are typically too burdensome for use in the Sustainment phase; however, the conventional self-report uniform estimate approaches could be pragmatic in this phase, as well as the semi-automated or automated EHR-based approaches. In contrast, during the pre-implementation planning phase of an EBP, estimation may be the only possible approach available if decision makers need data on the time required for alternate implementation strategies before these tasks have been pilot-tested. In sum, advances are needed in terms of highly rapid, rigorous, and low-resource time capture approaches, and the semi-automated and automated approaches described here provide innovative steps forward towards that goal.

Strengths of this report include its summary of key emerging EHR-based semi-automatic and automatic approaches to capturing time and the concrete case study examples (Table 3). Further, the 5 R’s model provided a systematic basis on which to evaluate the pragmatism of different approaches. In addition, reporting staff time as a cost is consistent with the recommendations from the Consolidated Health Economic Evaluation Reporting Standards (CHEERS) [29] to “describe the methods for valuing each resource in terms of its unit cost.” However, depending on the EHR approach used, the automated approach may be challenged to separately report the distinct resource costs for the intervention and the implementation strategy, as others have recommended [5]. Although beyond the scope of this brief review, those applying these different approaches to staff time estimation should keep in mind the CHEERS recommendations to specify which staff are included (e.g., clinical staff, contracted coaches) and from what perspective (e.g., clinical health system staff, research staff) [29].

Limitations include that our focused environmental scan on conventional self-report/observation approaches to staff time estimation and EHR-based semi-automatic and automatic approaches did not include all potential approaches relevant for IS, such as automated assessments by radiofrequency identification (RFID) tags or readers. In addition, our comparisons according to the 5 R’s model are necessarily subjective. A future systematic review would expand and add rigor to this environmental scan. Automated EHR-based approaches have been used internally by health systems more often than in IS research; thus, there are some key limitations in terms of sparse prior reporting of details and validation of these approaches [25]. However, some of the described EHR approaches have been validated against direct observation and demonstrated that > 80% of the time the estimates are within 3 min of each other [27]. When used for research, it is reasonable to initially vet the accuracy of automated EHR-based approaches as compared to observation [25], as was done in case example 2—this is particularly important for complex processes that are prone to interruptions.

## Conclusions

We summarized the strengths and limitations of different conventional and EHR-based semi-automated and automated approaches to measuring staff time as a cost for IS studies, with an emphasis on the 5 R’s model as an index of factors that are important to stakeholders. This is critical to allow decision-makers to consider the feasibility of implementing and sustaining programs, based on the estimates of staff time required. Going forward, the field should continue to identify additional methods of estimating staff time (and other implementation costs) that are rigorous and replicable, and also relevant, rapid, and low-resource enough to be measured in a EBP sustainment phase.

## Data Availability

Not applicable.

## Abbreviations

CHEERS: Consolidated Health Economic Evaluation Reporting Standards
EHR: Electronic health record
EBP: Evidence-based programs
IS: Implementation science
TDABC: Time-driven activity-based costing
VA: Veteran’s Affairs
5 R’s model: Relevance, rapidity, rigor, resources, and replicability model

## References

[1] Kilbourne AM, Elwy AR, Sales AE, Atkins D (2017). Accelerating research impact in a learning health care system: VA’s quality enhancement research initiative in the choice act era. Med Care.

[2] Aarons GA, Sklar M, Mustanski B, Benbow N, Brown CH (2017). “Scaling-out” evidence-based interventions to new populations or new health care delivery systems. Implement Sci.

[3] Kaplan RS, Witkowski M, Abbott M, Guzman AB, Higgins LD, Meara JG (2014). Using time-driven activity-based costing to identify value improvement opportunities in healthcare. J Health Manag.

[4] Keel G, Savage C, Rafiq M, Mazzocato P (2017). Time-driven activity-based costing in health care: a systematic review of the literature. Health Policy.

[5] Cidav Z, Mandell D, Pyne J, Beidas R, Curran G, Marcus S (2020). A pragmatic method for costing implementation strategies using time-driven activity-based costing. Implement Sci.

[6] Kilbourne AM, Goodrich DE, Miake-Lye I, Braganza MZ, Bowersox NW (2019). Quality enhancement research initiative implementation roadmap: toward sustainability of evidence-based practices in a learning health system. Med Care.

[7] Roberts SLE, Healey A, Sevdalis N (2019). Use of health economic evaluation in the implementation and improvement science fields-a systematic literature review. Implement Sci.

[8] Smith JD, Hasan M (2020). Quantitative approaches for the evaluation of implementation research studies. Psychiatry Res.

[9] Eisman AB, Kilbourne AM, Dopp AR, Saldana L, Eisenberg D (2020). Economic evaluation in implementation science: making the business case for implementation strategies. Psychiatry Res.

[10] Wagner TH, Yoon J, Jacobs JC, So A, Kilbourne AM, Yu W (2020). Estimating costs of an implementation intervention. Med Decis Making.

[11] Peek CJ, Glasgow RE, Stange KC, Klesges LM, Purcell EP, Kessler RS (2014). The 5 R’s: an emerging bold standard for conducting relevant research in a changing world. Ann Fam Med.

[12] Proctor EK, Powell BJ, McMillen JC (2013). Implementation strategies: recommendations for specifying and reporting. Implement Sci.

[13] Glasgow RE (2013). What does it mean to be pragmatic? Pragmatic methods, measures, and models to facilitate research translation. Health Educ Behav.

[14] Hsieh HF, Shannon SE (2005). Three approaches to qualitative content analysis. Qual Health Res.

[15] Ritzwoller DP, Glasgow RE, Sukhanova AY, Bennett GG, Warner ET, Greaney ML (2013). Economic analyses of the Be Fit Be Well program: a weight loss program for community health centers. J Gen Intern Med.

[16] Hoeft TJ, Wilcox H, Hinton L, Unützer J (2019). Costs of implementing and sustaining enhanced collaborative care programs involving community partners. Implement Sci.

[17] Kim KE, Tangka FKL, Jayaprakash M, Randal FT, Lam H, Freedman D (2020). Effectiveness and cost of implementing evidence-based interventions to increase colorectal cancer screening among an underserved population in Chicago. Health Promot Pract.

[18] Jordan N, Graham AK, Berkel C, Smith JD (2019). Costs of preparing to implement a family-based intervention to prevent pediatric obesity in primary care: a budget impact analysis. Prev Sci.

[19] Nguyen HN, Sammer MB, Bales B, Cano MC, Trout AT, Dillman JR (2020). Time-driven activity-based cost comparison of three imaging pathways for suspected midgut volvulus in children. J Am Coll Radiol.

[20] Collins CI, Hasan TF, Mooney LH, Talbot JL, Fouraker AL, Nelson KF (2020). Subarachnoid hemorrhage “fast track”: a health economics and health care redesign approach for early selected hospital discharge. Mayo Clin Proc Innov Qual Outcomes.

[21] Boyce-Fappiano D, Ning MS, Thaker NG, Pezzi TA, Gjyshi O, Mesko S (2020). Time-driven, activity-based cost analysis of radiation treatment options for spinal metastases. JCO Oncol Pract.

[22] Simeon K, Sharma M, Dorward J, Naidoo J, Dlamini N, Moodley P (2019). Comparative cost analysis of point-of-care versus laboratory-based testing to initiate and monitor HIV treatment in South Africa. PLoS One.

[23] Bobade RA, Helmers RA, Jaeger TM, Odell LJ, Haas DA, Kaplan RS (2019). Time-driven activity-based cost analysis for outpatient anticoagulation therapy: direct costs in a primary care setting with optimal performance. J Med Econ.

[24] Laviana AA, Ilg AM, Veruttipong D, Tan HJ, Burke MA, Niedzwiecki DR (2016). Utilizing time-driven activity-based costing to understand the short- and long-term costs of treating localized, low-risk prostate cancer. Cancer.

[25] Sinsky CA, Rule A, Cohen G, Arndt BG, Shanafelt TD, Sharp CD (2020). Metrics for assessing physician activity using electronic health record log data. J Am Med Inform Assoc.

[26] Huebschmann AG, Glasgow RE, Leavitt IM, Chapman K, Rice JD, Lockhart S, et al. Integrating a physical activity coaching intervention into diabetes care: a mixed methods evaluation of a pilot pragmatic trial. Transl Behav Med. 2022; in press. 10.1093/tbm/ibac014 PMC9150080 35312788

[27] Hribar MR, Read-Brown S, Goldstein IH, Reznick LG, Lombardi L, Parikh M (2018). Secondary use of electronic health record data for clinical workflow analysis. J Am Med Inform Assoc.

[28] Lopetegui M, Yen PY, Lai A, Jeffries J, Embi P, Payne P (2014). Time motion studies in healthcare: what are we talking about?. J Biomed Inform.

[29] Husereau D, Drummond M, Petrou S, Carswell C, Moher D, Greenberg D (2013). Consolidated Health Economic Evaluation Reporting Standards (CHEERS)--explanation and elaboration: a report of the ISPOR Health Economic Evaluation Publication Guidelines Good Reporting Practices Task Force. Value Health.

